# Prevalence, characteristics, and distribution of HPV genotypes in women from Zhejiang Province, 2016–2020

**DOI:** 10.1186/s12985-021-01676-z

**Published:** 2021-10-20

**Authors:** Xiaotian Yan, Lingwei Shen, Yufei Xiao, Qi Wang, Fugang Li, Yun Qian

**Affiliations:** 1 Department of Clinical Laboratory, Stomatology Hospital, School of Stomatology, Zhejiang University School of Medicine, Clinical Research Center for Oral Biomedical Research of Zhejiang Province, Cancer Center of Zhejiang University, Hangzhou, China; 2 Department of Clinical Laboratory, The Second Affiliated Hospital, Zhejiang University School of Medicine, Hangzhou, China; 3 Shanghai Upper Bio Tech Pharma Company Limited, Shanghai, China

**Keywords:** Human papillomavirus_1_, Genotype_2_, Cervical lesions_3_, Cervical cancer_4_, Vaccine_5_

## Abstract

**Objective:**

To evaluate and understand the prevalence of HPV genotypes and characteristics of female populations in specific areas and the relationship with cervical lesions, which can effectively guide cervical cancer screening and formulate HPV vaccine prevention strategies.

**Methods:**

A total of 77,443 women who visited gynecological clinics and underwent health examinations in the Second Affiliated Hospital of Zhejiang University School of Medicine during 2016–2020 were enrolled in this survey. Cervical samples were collected for HPV DNA genotyping and cervical cytology testing. Cervical biopsies were performed for patients with visible cervical abnormality or abnormal cytological results.

**Results:**

The results showed the 5-year overall positive rate was 22.3%, of which the gynecology clinic group had significantly more positive results compared with the health examination group (*P* < 0.001). The five most common genotypes in Zhejiang Province were HPV 52, 58, CP8304, 16, and 51 (23.9%, 12.7%, 11.7%, 11.7% and 9.3%). HPV infection was age-specific, with the highest infection rate in the age group ≤ 20 compared to other age groups (*P* < 0.001). HPV infection was also season-specific, with the highest infection rate in spring or winter. The main HPV infection mode was single infection (*P* = 0.004), but patients ≤ 20 years old were more likely to develop multiple infections (51.0%). HPV 16, 52 and 58 were the main genotypes that caused cytological abnormalities and HPV16, 18, 56, 58 and 66 were independent risk factors for cervical lesions (OR = 2.352, 1.567, 2.000, 1.694, 1.889; all *P* < 0.05). Further analysis found HPV 16 and 18 were the main genotypes that cause cervical cancer histological abnormalities and were independent risk factors for cervical cancer (OR = 5.647, *P* < 0.001; OR = 3.495, *P* = 0.036).

**Conclusion:**

This article analyzed the prevalence of distribution characteristics of HPV infection and revealed the corelation between HPV infection and cytological and histological abnormalities. Comprehensive results of this survey will help Zhejiang Province to formulate public health policies and provide evidence for future selection of specific HPV vaccines.

## Introduction

As the fourth most common cancer among women worldwide [1], cervical cancer is a significant threat to women. Globally, there are more than 500,000 new cases of cervical cancer each year. Approximately 250,000 women die from cervical cancer every year [2].

Persistent human papillomavirus (HPV) infection is a significant factor in causing cervical cancer [3]. At present, more than 200 types of HPV have been identified. According to the pathogenicity, HPV invades the genital tract and is usually categorized as high-risk (HR) or low-risk (LR) genotypes. It is reported that more than 15 HR-HPV genotypes are linked with the development of cervical cancer. HPV 16, 18, 31, 33, 35, 45, 52, and 58 are related to 90% of invasive cervical cancer [4]. Additionally, LR-HPV genotypes, including HPV 6, 11, 42, 43, and 44 are related to proliferative lesions [5, 6].

However, cervical cancer is a highly preventable and curable cancer; primary prevention and screening are the most effective ways to release the burden of HPV infection and reduce the mortality of cervical cancer. It is noteworthy that the promotion of the HPV vaccine has made a significant contribution to the prevention of cervical cancer [7–9]. The HPV vaccine currently implemented in Zhejiang Province includes bivalent, quadrivalent, and 9-valent vaccines. The 9-valent HPV vaccine targets HPV 6, 11, 16, 18, 31, 33, 45, 52, and 58, and potentially offers 90% protection against cervical cancer [10].

Due to differences in climate, living habits, and population distribution, different regions show different infection rates of HPV in China [11, 12]. Understanding the prevalence and distribution of HPV genotypes in a specific area is of great significance for guiding the use of cervical cancer vaccines in the area and formulating cervical cancer prevention and treatment strategies. There are currently several studies that have observed the HPV epidemiology in Zhejiang Province but the results vary significantly [13–15].

This article collected data from patients who have undergone HPV screening in the Second Affiliated Hospital of Zhejiang University School of Medicine’s health examination center and gynecological clinic between 2016 and 2020. The study analyzed the prevalence of HPV infection, the distribution of genotypes, and the characteristics of age-specific and season-specific infections. At the same time, the association of HPV infection with histological and cytological results were observed. This study aimed to provide a reference for the early prevention, detection, and control of cervical cancer, and a guide for the promotion and application of targeted vaccines for women in Zhejiang Province.

## Methods

### Patients and enrollment criteria

The Second Affiliated Hospital of Zhejiang University School of Medicine, as a well-known top hospital in the capital city of Zhejiang Province and one of the designated hospitals for cervical cancer screening, has huge daily outpatient visits and diverse patients, has huge daily outpatient visits and diverse patients. In this retrospective study, in order to obtain more accurate and reliable data, we collected large-scale data on female patients who attended health examination center and gynecological outpatient clinics of the Second Affiliated Hospital of Zhejiang University School of Medicine during 2016–2020. All patients were divided into two groups: health examination group (HEG) and gynecological clinic group (GCG). This study was approved by the Ethics Committee of the Second Affiliated Hospital of Zhejiang University School of Medicine. The review committee waived the need of patients’ consent due to anonymous analyses of the data. The management and publication of patient information in this study was strictly in accordance with the Declaration of Helsinki, including the confidentiality and anonymity.

Inclusion criteria: (1) history of sexual activity at any age; (2) voluntary HPV test; (3) had not had sexual intercourse, nor used vaginal drugs in the previous 48 h; and (4) had not been vaccinated against HPV.

Exclusion criteria: (1) patients with other infections or autoimmune diseases; (2) pregnant women; (3) patients who had undergone cervical surgery or hysterectomy; and (4) patients who had undergone immunosuppressive therapy.

### Cervical samples collection

The clinician used a speculum to expose the cervix and wipe off excessive secretions with a cotton swab. A cervical brush was rotated slowly in one direction for 6–7 times to obtain sufficient cervical epithelial cells; this was repeated twice. One of the cytology brushes was used for HPV-DNA detection, and the other brush was placed in preservation solution for a ThinPrep cytologic test (TCT, Hologic, USA, MA). All samples were stored at 4 ℃ and tested within 48 h.

### HPV DNA genotyping

The PCR-reverse dot blot hybridization test was used for HPV DNA genotyping detection. The YN-H16 constant temperature hybridization instrument, cervical exfoliated cell collector, and HPV genotyping detection kit were provided by 21 HPV GenoArray Diagnostic Kit (HBGA-21PKG) (Hybribio, China, Guangdong) and GenPlex® microfluidic automatic nucleic acid detection (Type 24, Bohui, China, Beijing). HPV DNA extraction, PCR amplification, hybridization, and result interpretation were performed according to the manufacturer’s instructions.

It is worth noting that the position where the blue spots appear on the membrane strip relates to genotype information marked on the corresponding position. If only one genotype site has blue spots, it indicates a single infection of the corresponding genotype; if multiple genotype sites have blue spots, it indicates a mixed infection of the corresponding genotypes.Two types of HPV genotype tests were carried out clinically, HPV-DNA 21 and HPV-DNA 24, and included 18 high-risk HPV types (16, 18, 31, 33, 35, 39, 45, 51, 52, 53, 56, 58, 59, 66, 68, 73, 82, and 83), and seven low-risk HPV types (6, 11, 42, 43, 44, 81, and CP8304) [16].

### ThinPrep cytologic test

Cervical slides were analyzed with TCT and diagnosed by two experienced pathologists. The TCT results were classified according to the Bethesda 2001 criteria [17], including negatives of intraepithelial lesions or malignancy (NILM); Atypical glandular cells (AGC); atypical squamous cells high-grade (ASC-H); atypical squamous cells, not excluding high-grade squamous intraepithelial lesion (ASC-US); low-grade squamous intraepithelial lesion (LSIL); high-grade squamous intraepithelial lesion (HSIL); and invasive cervical cancer (ICC).

### Histology diagnosis

A cervical biopsy was performed for patients with visible cervical abnormality or abnormal cytological results. The histology results were graded by two professional pathologists according to the pathological diagnosis standard criteria, including no lesions, cervical intraepithelial neoplasia (CIN 1, CIN 2, CIN 3), and cervical cancer.

### Statistical analysis

All statistical analyses were performed using R software 3.5.1, Excel 2019 and SPSS 22.0 software (SPSS Inc., Chicago, IL, USA). Numerical data were expressed as a percentage (%). Pearson's χ2 test and binary logistic regression analysis were performed to evaluate the significance of the difference between the specified groups. *P* < 0.05 was considered statistically significant.

## Results

### Prevalence of HPV infection in HEG and GCG

In total, data was collected from 77,443 HPV screening tests from the HEG (n = 29,216) and GCG (n = 48,227) between 2016 and 2020. Among them, 17,234 HPV-positive patients were screened and the average infection rate over 5 years was 22.3%. The annual HPV infection rates in HEG were 13.2%, 13.6%, 15.3%, 13.6%, and 12.4%, and the 5-year average infection rate was 13.6 ± 0.9% (Table 1). The annual HPV infection rates in GCG were 23.7%, 27.1%, 28.5%, 28.6%, and 27.4%, and the 5-year average infection rate was 27.0 ± 1.8% (Table 1). The HPV infection rate in the GCG was significantly higher than HEG (*P* < 0.001).

**Table1:** The HPV infection rate of gynecology clinic group and health examination group, 2016–2020

	2016	2017	2018	2019	2020	Five years
	No. (%)	No. (%)	No. (%)	No. (%)	No. (%)	No. (%)
GCG	1470 (23.7)	2107 (26.0)	2181 (28.5)	4817 (28.6)	2680 (27.4)	13,255 (27.3)
HEG	663 (13.2)	944 (13.6)	687 (13.6)	1153 (13.6)	532 (12.4)	3979 (13.6)
Total	2133 (19.0%)	3051 (20.1%)	2868 (23.6%)	5970 (23.6%)	3212 (22.8%)	17,234 (22.3%)

% = the number of HPV-positive patients/the number of total patients in specific group during specific year * 100%

### Prevalence of HPV infection change by year and season

**Fig. 1:**
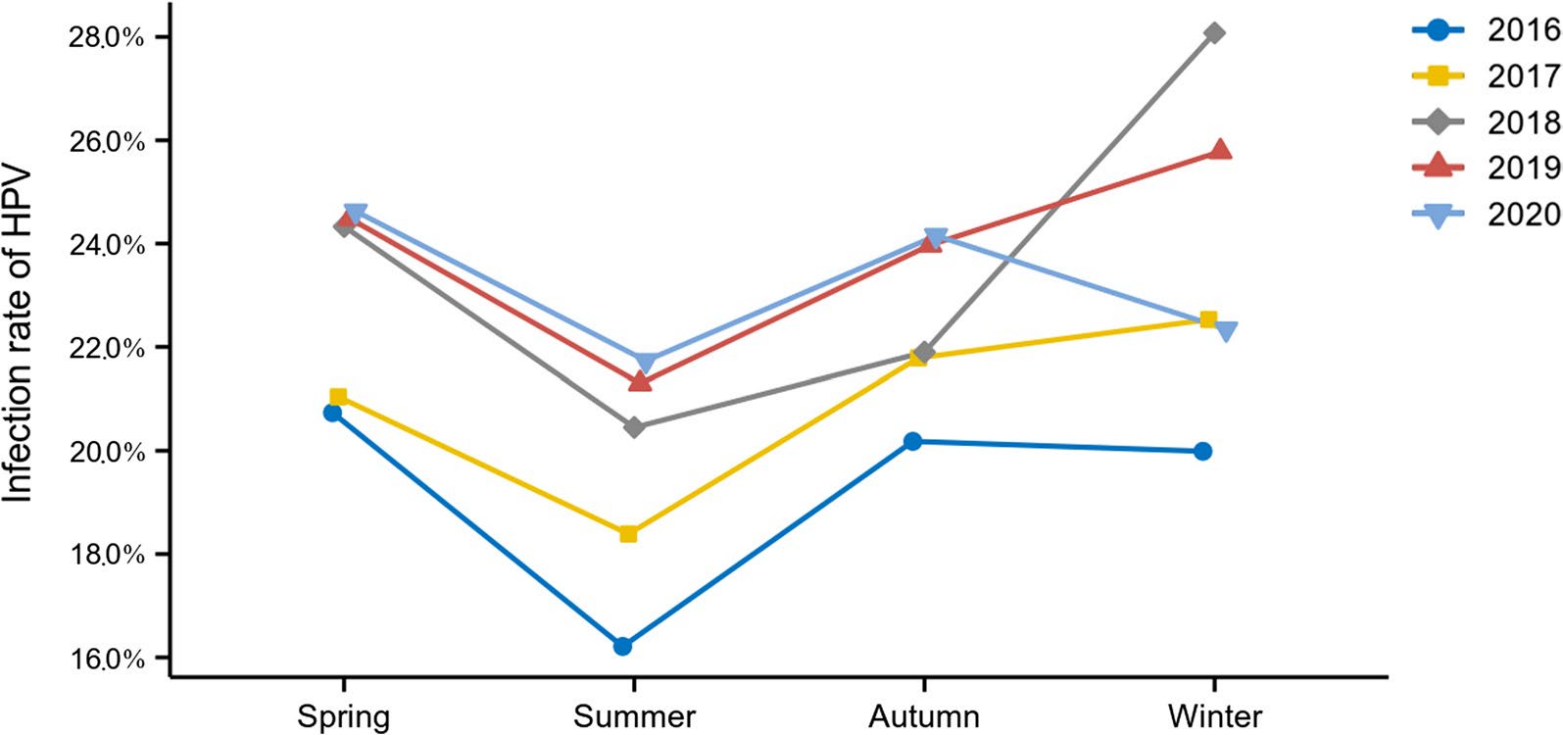
The prevalence of HPV infection rate over different seasons from 2016 to 2020. The HPV infection rate in summer was the lowest; the HPV infection rate in winter or spring was the highest during the 2016–2020

The change trend of HPV infection rate from 2016 to 2020 in HEG and GCG can be observed by comparing the data in Table 1 horizontally. The infection rate in HEG was generally stable during the 5-year period while the GCG and overall infection rate demonstrated an upward trend each year. In addition, the infection rates over different seasons of each year were also analyzed, as shown in Fig. 1. Despite the difference of infection rates between each season was not significant (*P* = 0.063), it can be seen that during 2016–2020, the HPV infection rate in summer was the lowest; the HPV infection rate in winter or spring was the highest.

### Distribution of HPV genotypes in HEG and GCG

**Fig. 2:**
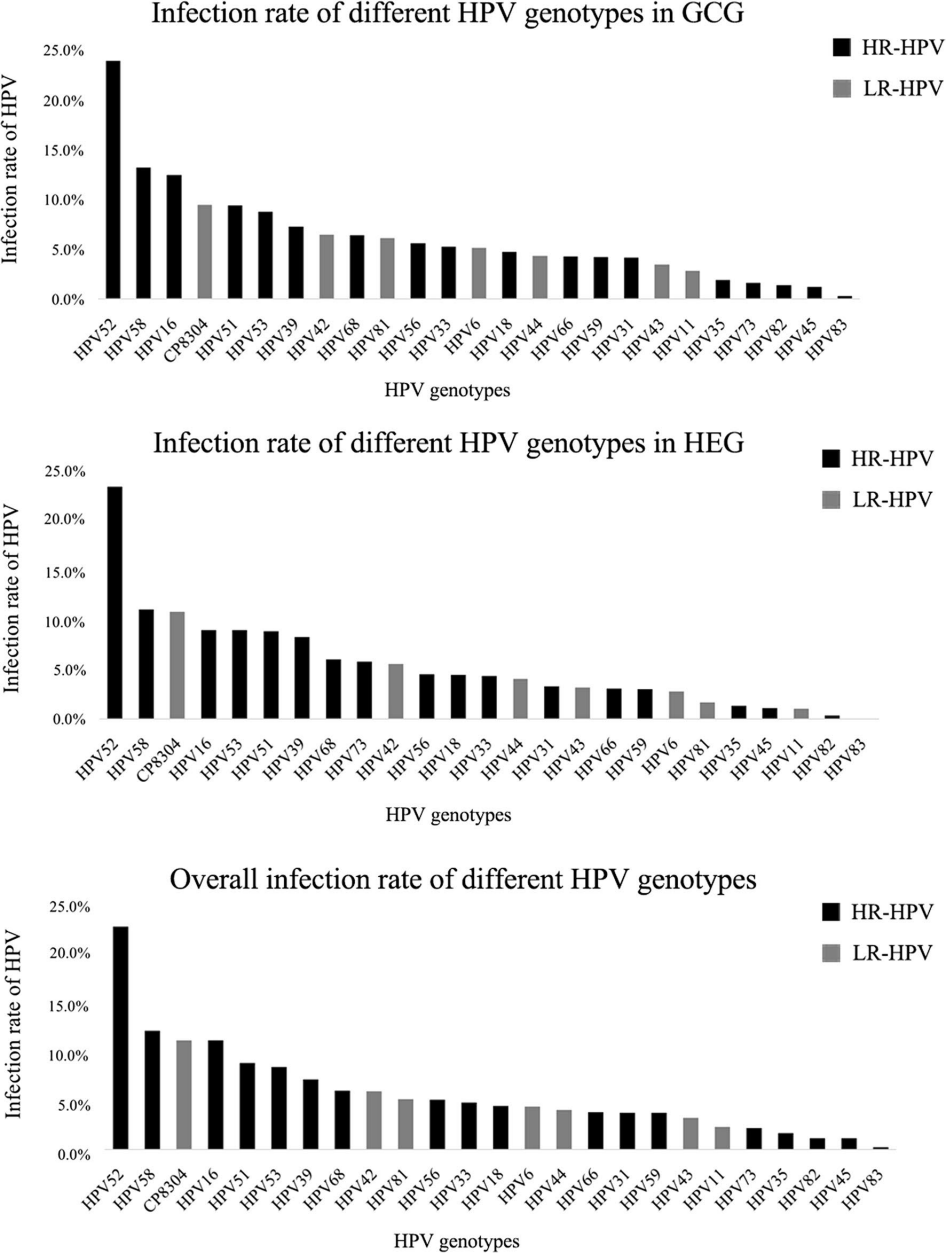
Distribution of HPV genotypes over the past 5 years. The distribution of infection rate for each genotype in the past 5 years is shown in Fig. 2. HPV 52 and 58 were two most common genotypes

The distribution of infection rate for each genotype over the 5-year period is shown in Fig. 2. Overall, the five genotypes with the highest positive rate were HPV 52 (23.9%), 58 (12.7%), CP8304 (11.7%), 16 (11.7%), and 51 (9.3%).

In HEG, the top five genotypes were HPV 52, 58, CP8304, 16, and 53, and the positive rates were 23.5%, 11.0%, 10.8%, 9.0% and 9.0% respectively. In GCG, the top five genotypes were HPV 52, 58, 16, CP8304 and 51, and the infection rates were 24.0%, 13.2%, 12.5%, 9.5%, and 9.5%.

### Prevalence of HPV grouped by age in study population

**Fig. 3:**
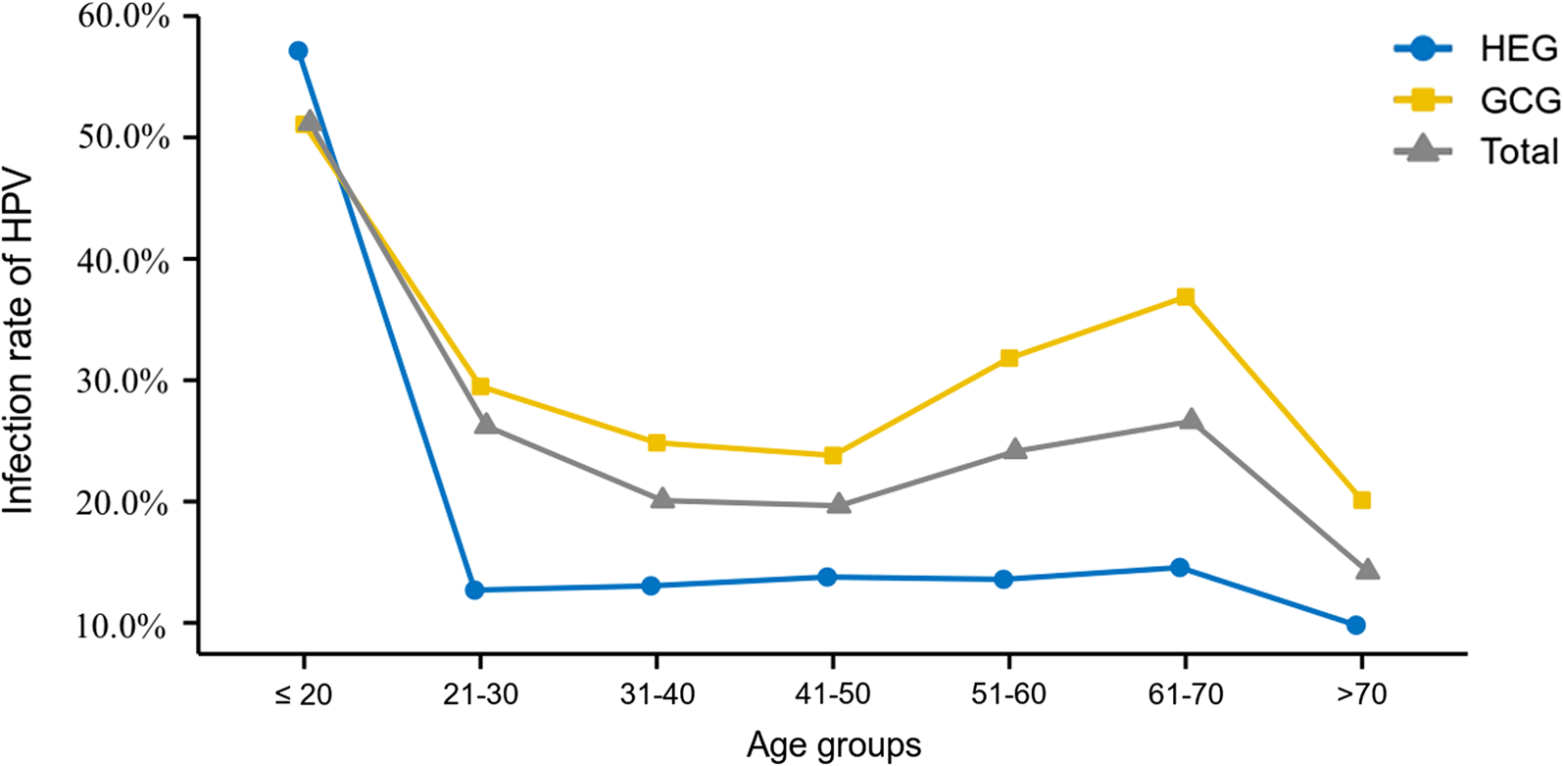
The overall age-specific HPV infection rate over the past 5-years. The HPV infection rate in different age groups is shown in Fig. 3. The ≤ 20 age group had the highest infection rate and the age group 61–70 ranked second

Among the 77,443 patients included in this study, patient’s ages ranged from 10 to 98 years old with an average of 41 ± 11 years. All patients were divided into seven age groups (≤ 20, 21–30, 31–40, 41–50, 51–60, 61–70, and > 70). HPV infection rates in different age groups were analyzed as shown in Fig. 3. The overall age-specific prevalence curve revealed two peaks. The first peak appeared in the ≤ 20 group and the infection rate was 51.2%. As the age increased, the infection rate decreased, until the second peak occurred in the age group 61–70 and the infection rate was 26.6%. The age-specific prevalence curve of HEG and GCG also showed two peaks at the ≤ 20 and 61–70 age groups. In addition, the HPV infection rate in ≤ 20 age group was significantly higher than that in other groups (*P* < 0.001).

### Distribution of HPV genotypes grouped by age in HPV-positive population

We further evaluated the positive rate of each genotype in each age group to explore the age-specific distribution of genotypes.

**Fig. 4:**
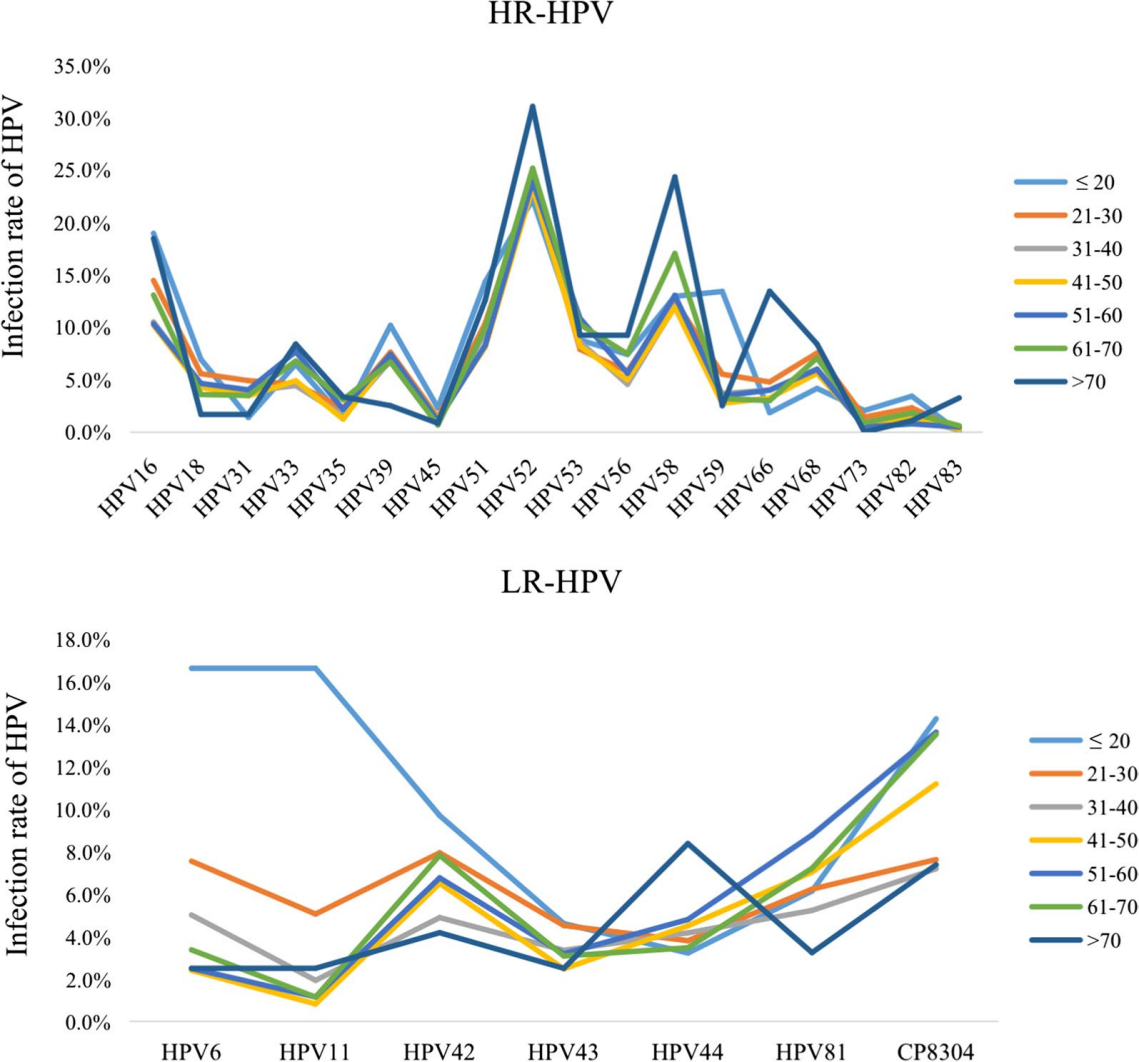
HR-HPV and LR-HPV distribution in different age groups during the past 5 years. Among HR HPV, HPV 52 has the highest positive rate. Among LR HPV, CP8304 has the highest positive rate in most age groups, except the ≤ 20 and > 70 group

It can be observed that, regardless of age group, HPV 52 had the highest positive rate (*P* < 0.05). The distribution of HR-HPV and LR-HPV in different age groups is shown in Fig. 4. Among HR-HPV, HPV 58 ranked second in most age group. In the age groups of ≤ 20 and 21–30, HPV 16 ranked second. Among LR HPV, CP8304 had the highest positive rate in most age groups, except the ≤ 20 and > 70 groups.

### Prevalence of HPV single and multiple HPV genotypes infection

Table 2 showed the distribution characteristics of HPV infection type in different age groups. Single infections were the most common infection pattern in all age groups (*P* = 0.004). The infection rate of single infections reached its peak in the 41–50 group. Regarding multiple infections, the main manifestation was a double infection. The age group of ≤ 20 accounted for the largest proportion.

**Table 2:** Prevalence of single and multiple HPV infections in different age groups

	Single type infection	Double type infection	Triple type infection	Quadruple type infection	Fifth type infection and more
	No. (%)	No. (%)	No. (%)	No. (%)	No. (%)
≤ 20	106 (49.1)	55 (25.5)	30 (13.9)	14 (6.5)	11 (5.1)
21–30	2581 (63.7)	929 (22.9)	352 (8.7)	118 (2.9)	74 (1.8)
31–40	3705 (76.1)	871 (17.9)	216 (4.4)	58 (1.2)	16 (0.3)
41–50	3193 (79.0)	659 (16.3)	135 (3.3)	43 (1.1)	14 (0.4)
51–60	2080 (71.7)	567 (19.5)	175 (6.0)	49 (1.7)	32 (1.1)
61–70	687 (66.6)	223 (21.6)	81 (7.9)	28 (2.7)	13 (1.3)
> 70	74 (62.2)	20 (16.8)	10 (8.4)	10 (8.4)	4 (3.4)
Total	12,426 (72.1)	3324 (19.3)	999 (5.8)	320 (1.9)	164 (1.0)

It is worth noting that in the age group ≤ 20, the probability of multiple infections (double + triple + quadruple + fifth infections) was higher than single infection, which accounted for more than 50%.

### Distribution of HPV genotypes according to cytology abnormalities

**Fig. 5:**
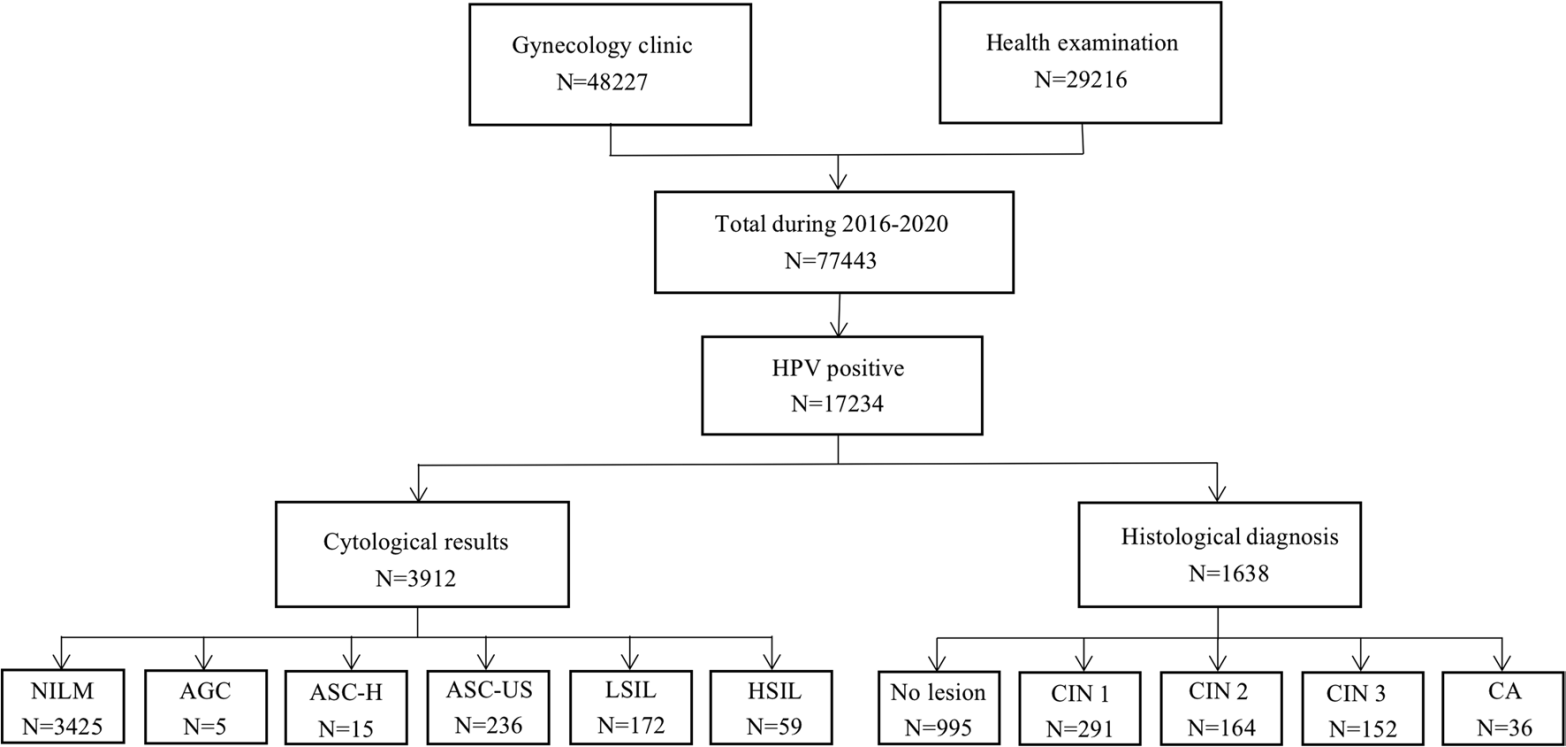
Flow chart of the study samples. The process of obtaining cytological test results and pathological biopsy specimens is shown in Fig. 5

**Fig. 6:**
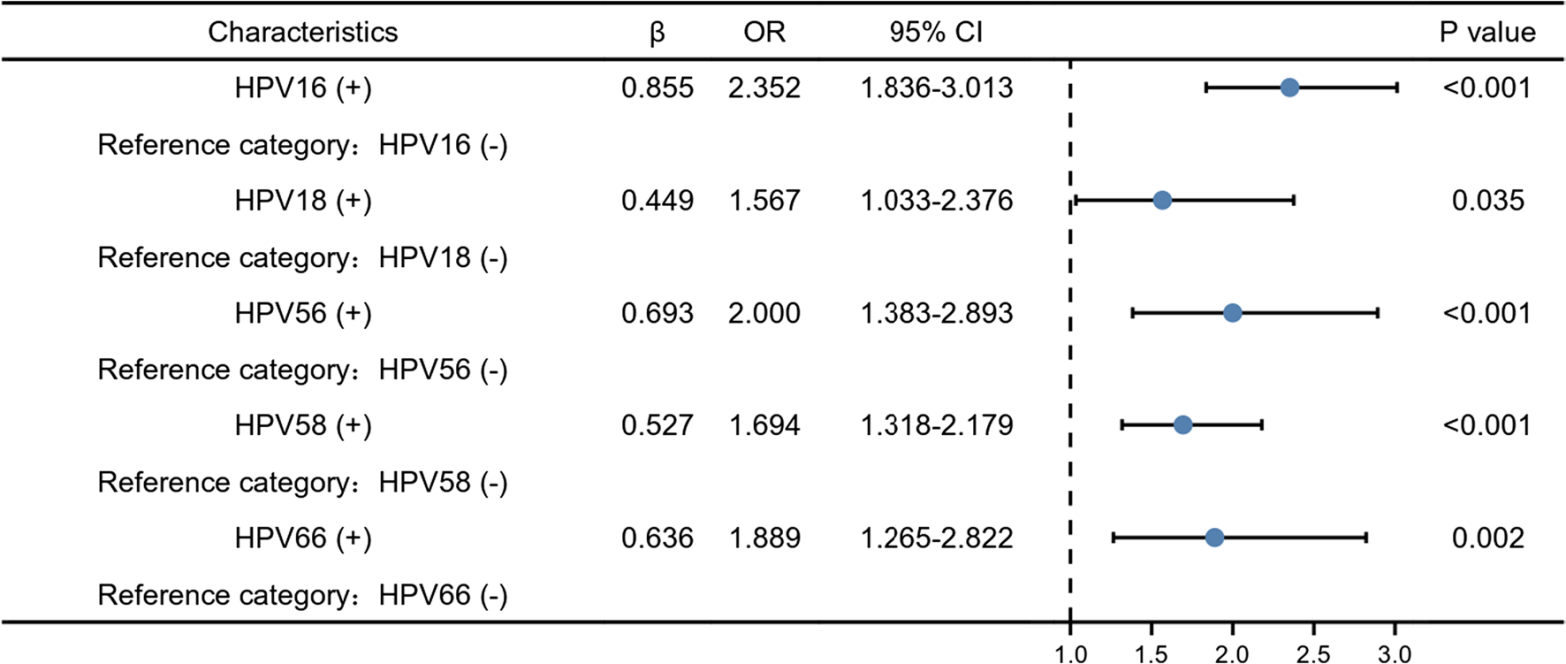
Multivariate logistic regression analysis of risk factors for cervical cytology abnormalities. Odds ratios with 95% confidence intervals of HPV genotypes associated with abnormal cervical cytology were provided. HPV16, 18, 56, 58 and 66 were independent risk factors for abnormal cervical cytology. Other HPV genotypes showed no significant differences in abnormal specimens from normal specimens and no OR was listed

The process of obtaining cytological test results and pathological biopsy specimens is shown in Fig. 5. For further screening of 17,234 positive HPV patients, the cytology results from 3912 patients were analyzed. The distribution of HPV genotypes in different cytology results is shown in Table 3. Among them, 487 patients demonstrated abnormal cytology (12.5%). The results suggest that HPV52, 16, and 58 are the three main genotypes that cause cytological abnormalities, regardless of the stage of cervical lesions. In addition, the results of multivariate logistic regression analysis are shown in Fig. 6. HPV16, 18, 56, 58 and 66 were independent risk factors for abnormal cervical cytology (OR = 2.352, 1.567, 2.000, 1.694, 1.889; all *P* < 0.05).

**Table 3:** Distribution of HPV genotypes according to cytology abnormalities

	NILM	AGC	ASC-H	ASC-US	LSIL	HSIL
	No. (%)	No. (%)	No. (%)	No. (%)	No. (%)	No. (%)
HR-HPV						
HPV16	379 (11.1)	2 (40.0)	6 (40.0)	37 (15.7)	34 (19.8)	24 (40.7)
HPV18	152 (4.4)	0 (0.0)	0 (0.0)	13 (5.5)	7 (4.1)	8 (13.6)
HPV31	141 (4.1)	0 (0.0)	0 (0.0)	12 (5.1)	4 (2.3)	5 (8.5)
HPV33	198 (5.8)	1 (20.0)	4 (26.7)	11 (4.7)	9 (5.2)	7 (11.9)
HPV35	60 (1.8)	1 (20.0)	0 (0.0)	1 (0.4)	2 (1.2)	2 (3.4)
HPV39	274 (8.0)	0 (0.0)	1 (6.7)	1 4(5.9)	7 (4.1)	1 (1.7)
HPV45	52 (1.5)	1 (20.0)	1 (6.7)	5 (2.1)	0 (0.0)	1 (1.7)
HPV51	306 (8.9)	0 (0.0)	0 (0.0)	23 (9.8)	25 (14.5)	1 (1.7)
HPV52	854 (24.9)	1 (20.0)	5 (33.3)	65 (27.5)	39 (22.7)	7 (11.9)
HPV53	313 (9.1)	0 (0.0)	2 (13.3)	21 (8.9)	22 (12.8)	0 (0.0)
HPV56	163 (4.8)	0 (0.0)	0 (0.0)	19 (8.1)	22 (12.8)	0 (0.0)
HPV58	463 (13.5)	2 (40.0)	4 (26.7)	45 (19.1)	32 (18.6)	10 (17.0)
HPV59	143 (4.2)	0 (0.0)	0 (0.0)	12 (5.1)	10 (5.8)	0 (0.0)
HPV66	230 (6.7)	1 (20.0)	1 (6.7)	14 (5.9)	16 (9.3)	0 (0.0)
HPV68	225 (6.6)	1 (20.0)	0 (0.0)	17 (7.2)	7 (4.1)	0 (0.0)
HPV73	18 (1.0)	0 (0.0)	0 (0.0)	0 (0.0)	0 (0.0)	0 (0.0)
HPV82	21 (1.1)	0 (0.0)	0 (0.0)	1 (0.8)	1 (1.2)	0 (0.0)
HPV83	6 (0.3)	0 (0.0)	0 (0.0)	2 (1.7)	0 (0.0)	0 (0.0)
LR-HPV						
HPV6	118 (3.5)	0 (0.0)	1 (6.7)	11 (4.7)	6 (3.5)	1 (1.7)
HPV11	63 (1.8)	0 (0.0)	0 (0.0)	4 (1.7)	6 (3.5)	0 (0.0)
HPV42	222 (6.5)	0 (0.0)	0 (0.0)	18 (7.6)	15 (8.7)	1 (1.7)
HPV43	95 (2.8)	0 (0.0)	0 (0.0)	11 (4.7)	3 (1.7)	0 (0.0)
HPV44	162 (4.7)	0 (0.0)	0 (0 .0)	5 (2.1)	3 (1.7)	1 (1.7)
HPV81	124 (6.5)	0 (0.0)	0 (0.0)	6 (5.0)	5 (6.0)	1 (4.0)
CP8304	136 (8.9)	0 (0.0)	0 (0.0)	10 (8.6)	5 (5.7)	1 (3.0)

### Distribution of HPV genotypes according to histological abnormalities

A total of 1637 HPV-positive patients were pathologically diagnosed after vaginal biopsy. Among them, 642 cases had histological abnormalities (39.2%).

**Fig. 7:**
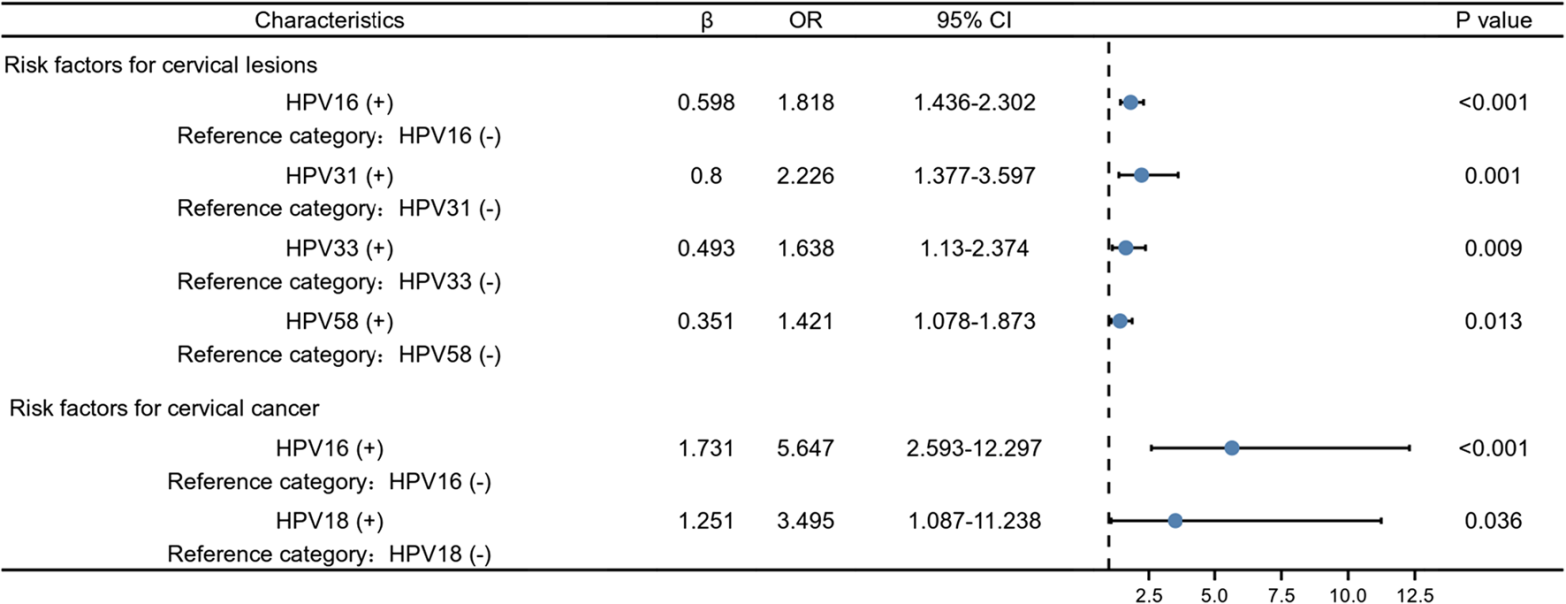
Multivariate logistic regression analysis of risk factors for cervical histological abnormalities. Odds ratios with 95% confidence intervals of HPV genotypes associated with abnormal cervical histology were provided. HPV16, 31, 33 and 58 were independent risk factors for the occurrence of cervical lesions. HPV16 and 18 were independent risk factors for disease progression from cervical intraepithelial neoplasia to cervical cancer

The distribution of HPV genotypes in different histology results is shown in Table 4. Among the 291 CIN 1, HPV 52 was the dominant genotype (27.5%), followed by HPV 16 (17.2%), and HPV 58 (15.8%). Among the 164 CIN 2, HPV 16 was the dominant genotype (31.1%), followed by HPV 52, and HPV 58 (both 26.6%). Among the 152 CIN 3, HPV 16 was the dominant genotype (46.1%), followed by HPV 52 (19.7%), and HPV 58 (15.8%). Among the 36 cervical cancer results, HPV 16 (58.3%) and 18 (22.2%) were the main genotypes. It was observed that as the degree of cervical lesions worsen, the infection rate of HPV16 increases (from 17.2 to 58.3%). As shown in Fig. 7, multivariate logistic regression analysis showed that patients infected with HPV 16, 31, 33 or 58 have a significantly increased risk of occuring cervical lesions (including cervical intraepithelial neoplasia and cervical cancer) (OR = 1.818, *P* < 0.001; OR = 2.226, *P* = 0.001; OR = 1.638, *P* = 0.009; OR = 1.421, *P* = 0.013); patients infected with HPV16 or 18 are at increased risk of developing cervical cancer from cervical epithelial neoplasia (OR = 5.647, *P* < 0.001; OR = 3.495, *P* = 0.036).

**Table 4:** Distribution of HPV genotypes according to histological abnormalities

	No lesions	CIN 1	CIN 2	CIN 3	Cervical cancer
	No. (%)	No. (%)	No. (%)	No. (%)	No. (%)
HR-HPV					
HPV16	197 (19.8)	50 (17.2)	51 (31.1)	70 (46.1)	21 (58.3)
HPV18	88 (8.8)	19 (6.5)	13 (6.5)	5 (3.3)	8 (3.3)
HPV31	32 (3.2)	17 (5.8)	14 (5.8)	8 (5.3)	2 (5.3)
HPV33	64 (6.4)	18 (6.2)	19 (11.6)	24 (15.8)	3 (15.8)
HPV35	19 (1.9)	7 (2.4)	5 (3.0)	3 (2.0)	0 (0.0)
HPV39	83 (8.3)	19 (6.5)	7 (4.3)	6 (3.9)	1 (3.9)
HPV45	10 (1.0)	5 (1.7)	2 (1.2)	1 (0.7)	1 (0.7)
HPV51	89 (8.9)	42 (14.4)	20 (12.2)	9 (5.9)	0 (0.0)
HPV52	246 (24.7)	79 (27.1)	42 (25.6)	30 (19.7)	1 (19.7)
HPV53	107 (10.8)	29 (10.0)	15 (9.1)	3 (2.0)	0 (0.0)
HPV56	70 (7.0)	19 (6.5)	10 (6.1)	5 (3.3)	0 (0.0)
HPV58	143 (14.4)	46 (15.8)	42 (25.6)	24 (15.8)	3 (15.8)
HPV59	39 (3.9)	11 (3.8)	7 (4.3)	3 (2.0)	0 (0.0)
HPV66	45 (4.5)	14 (4.8)	12 (7.3)	5 (3.3)	0 (0.0)
HPV68	67 (6.7)	17 (5.8)	7 (4.3)	3 (2.0)	1 (2.8)
HPV73	3 (0.6)	2 (1.4)	1 (1.2)	0 (0.0)	0 (0.0)
HPV82	5 (0.9)	3 (2.1)	0 (0.0)	1 (1.4)	1 (5.0)
HPV83	5 (0.9)	1 (0.7)	0 (0.0)	0 (0.0)	0 (0.0)
LR-HPV					
HPV6	25 (2.5)	10 (3.4)	2 (1.2)	1 (0.7)	0 (0.0)
HPV11	11 (1.1)	7 (2.4)	3 (1.8)	2 (1.3)	0 (0.0)
HPV42	47 (4.7)	18 (6.2)	10 (6.1)	6 (3.9)	2 (5.6)
HPV43	30 (3.0)	8 (2.7)	6 (3.7)	0 (0.0)	0 (0.0)
HPV44	39 (3.9)	9 (3.1)	8 (4.9)	4 (2.6)	0 (0.0)
HPV81	40 (7.4)	9 (6.3)	5 (5.8)	0 (0.0)	1 (5.0)
CP8304	33 (7.4)	10 (6.8)	5 (6.4)	5 (5.7)	0 (0.0)

## Discussion

In May 2018, the Director-General of the World Health Organization (WHO) called for action toward achieving the global elimination of cervical cancer [18]. As cervical cancer screening has not been carried out worldwide, the prevalence of the disease is still high [19].Therefore, early screening and timely treatment, as well as expanding vaccination coverage, are the main goals to improve the current situation. Determination of the type-specific HPV prevalence and distribution in specific areas are important parts of formulating prevention and control strategies to reduce the incidence of cervical cancer.

Among the participants in this study, the 5-year total HPV positive rate was 22.3%, similar to two other surveys carried out in Zhejiang Province [15, 20]. According to an HPV epidemiological study carried out among the general population in China (1.7 million), the five most common HPV types were HPV 16, 52, 58, 18, and 33 [21]. However, this study revealed that HPV 52, 58, CP8304, 16, and 53 were the most frequent genotypes, similar to the results of a study carried out in Guangdong Province [22]. Moreover, in this study, regardless of age group, HPV 52 was the genotype with the highest infection rate, followed by HPV 16 and 58 in HR-HPV, and CP8304 in LR-HPV. CP8304 as a type of HPV which Chinese people are susceptible to; the infection rate of CP8304 exceeds several HR-HPV genotypes. However, there are few studies focused on the prevalence of CP8304 and it is worthy of attention.

Since this study included patients from both the health examination center and gynecology clinic, it is a more representative reflection of the overall HPV infection situation. We found that the overall infection rate increased during the 5-year period, which may be due to increased awareness of prevention and the popularization of HPV screening, but it is also possible that, with the development of society and economy, young people's sexual habits have changed and they are sexually active earlier and have multiple partners.

Previous studies had shown that seasonal changes strongly affect biological functions, including susceptibility to infectious, metabolic, inflammatory, and malignant diseases [23, 24]. HPV infection and HPV induced cervical epithelial dysplasia and carcinomatous change may be novel sun exposure risks in summer [25]. Sunlight data was difficult for us to collect. Since the fluency of sunlight was related to seasonal changes, we evaluated the changes in HPV infection rate in different seasons each year. We found that the infection rate was higher in spring and winter but lower in summer in Zhejiang Province, similar to the results found in Guizhou [26]. However, the infection rate was highest in spring and summer in Kunming [27], and in Wuhan [28] the infection rate in summer and winter was higher than that in spring and autumn, which suggests that region and climate are factors that affect HPV infection rate. Although we were unable to provide a scientific explanation for the prevalence in a particular season, but this suggested that the season was an intervention factor related to HPV infection, and further investigations were needed.

A large number of studies have shown that HPV infection is significantly age-specific [15, 29]. The results of this study were in line with previous literature, and the distribution of HPV infection rate showed a bimodal pattern. We discovered young women under the age of 20 had the highest infection rate, possibly because they were new to sex and their immune systems had not been sensitized, were sensitive to HPV infections [30]. They were a high-risk group for HPV, but they generally presented with temporary infections. With an increase in age, HPV infection rate decreased, and there were second peaks of infection until ages 61–70. The reason may be due to the continued existence of the virus or the reactivation of potential HPV caused by physiological and immune disorders during the transitional period of menopause [31, 32]. Therefore, implementing a vaccine program before young people may become infected with HPV can effectively reduce the HPV infection rate. In addition, the HPV infection rate in women over 60 years old is increasing, so it is recommended that the maximum age for cervical cancer screening is raised.

The age-specific prevalence curve of a single HPV infection presented a unimodal distribution, reaching a peak in the group aged 41–50; the groups ≤ 20 years and > 70 years were quite low, which was similar to the findings by Shanxi and Beijing [33, 34]. Compared with other age groups, a larger proportion of people in the ≤ 20 years of age group presented with multiple HPV infections. Sexual behavior, viral characteristics, and host susceptibility may account for the prevalence differences [35]. Whether multiple infection genotypes increase the risk of cervical lesions has yet to be concluded. Studies have shown that the existence of multiple HPV genotypes may prolong the duration of HPV infection and may increase the risk of cancer [36]. Therefore, multiple infections are more dangerous than single infections. However, studies have also shown that there may be competition or counterbalance between various types of HPV [37], which affects the role of HPV in the progression of cervical cancer without increasing its incidence. This article did not study the correlation between the two to clarify the pathogenic mechanism and it is possible that the contribution of each genotype in cervical lesions may explain this correlation.

High-risk HPV infection is a primary factor in precancerous lesions and cervical cancer. We found that the distribution of high-risk HPV genotypes is directly related to the severity of cervical cytopathology. By analyzing the cytology results of 3,912 patients, it was found that regardless of AGC, ASC-H, ASC-US, LSIL, or HSIL cytology samples, HPV 16, 58, and 52 were always the major type, with variations in rank varied, which was consistent with the data in other studies [15, 38]. Furthermore, we noticed that patients infected with HPV 16, 18, 56, 58 or 66 have an increased risk of causing abnormal cytological results. Among them, the infection rate of HPV 56 and 66 was not high, but their role in developing cervical epithelial cell abnormalities is worthy of attention.

Among the population with abnormal histological results, the distribution of HPV was also different. But, in general, similar to the cytological results, we observed that in CIN 1, 2, and 3, HPV 16, 58, and 52 were always the major type. Additionally, with the increase of the histological lesions grade, the infection rate of HPV 16 also increased, especially in cervical cancer (58.33%). Multivariate logistic regression analysis showed HPV16 infection is closely associated with the severity of cervical intraepithelial neoplasia and cervical cancer. Moreover, HPV 18 was also the most common oncogenic type associated with cervical cancer, which was similar to previous studies [39]. Previous studies have shown that within one or two years after the initial infection, the persistence of high-risk HPV infection is highly predictive of the lifetime risk of pre-invasive and invasive cervical tumors. HPV genotype seems to be the most important factor of persistence, among which HPV 16 and HPV 18 are the most likely to persist [39–41].

It is our belief that the promotion and popularization of bivalent and quadrivalent vaccines has caused a decline in the infection rate of HPV 16 and 18. However, the overall HPV infection rate is still increasing; prevention of cervical cancer still has a long way to go. Although there are currently three preventive HPV vaccines (bivalent, quadrivalent, and 9-valent) in use globally, the WHO has not given biased recommendations for these three types of vaccines [42]. With regard to the valence of the vaccine, it is not the case that more is better, only highly targeted vaccines provide effective protection [43]. China should consider both cost-effectiveness and the actual type of HPV infection when selecting a suitable vaccine. Although Gardasil 9 targets many types of HPV, there are objective reasons, such as high price, low availability, and long appointment times, which result in very limited coverage of the population. Therefore, it is recommended that development of a domestic preventive HPV vaccine with independent property rights, based on HPV 16 and 18, covering HPV 52 and 58 according to the characteristics of the female HPV epidemic in China, would achieve primary prevention of cervical cancer.

There are some limitations in this study: First, due to sampling error, operation error, or other reasons, the results of the ThinPrep cytologic test may be over-diagnosed or missed. Second, the lack of detailed information on the geographic, socio-demographic, and behavioral characteristics of the study population prevents assessment of these characteristics on the HPV infection rate. Finally, the absence of data from other areas in Zhejiang Province means that these results might not be representative of all women in Zhejiang; a larger and more representative population is recommended for future research.

## Conclusion

In summary, this article analyzed the prevalence of HPV over 5 years and studied age distribution characteristics of different genotypes and single and multiple infection types. Moreover, the correlation between HPV infection and cervical lesions was also observed, which will help Zhejiang Province formulate public health policies and provide evidence for the future selection of specific HPV vaccines.

## Data Availability

All data generated or analyzed during this study are included in this published article.

## Abbreviations

HPV: Human papillomavirus
HR-HPV: High-risk HPV
LR-HPV: Low-risk HPV
TCT: ThinPrep cytologic test
NILM: Intraepithelial lesions or malignancy
AGC: Atypical glandular cells
ASC-H: Atypical squamous cells high-grade
ASC-US: Atypical squamous cells, not excluding high-grade squamous intraepithelial lesion
LSIL: Low-grade squamous intraepithelial lesion
HSIL: High-grade squamous intraepithelial lesion
ICC: And invasive cervical cancer
CIN: Cervical intraepithelial neoplasia
HEG: Health examination group
GCG: Gynecological clinic group
WHO: World Health Organization
